# Dynamic Modulation of Mouse Locus Coeruleus Neurons by Vasopressin 1a and 1b Receptors

**DOI:** 10.3389/fnins.2018.00919

**Published:** 2018-12-10

**Authors:** Elba Campos-Lira, Louise Kelly, Mohsen Seifi, Torquil Jackson, Torsten Giesecke, Kerim Mutig, Taka-aki A. Koshimizu, Vito S. Hernandez, Limei Zhang, Jerome D. Swinny

**Affiliations:** ^1^Departamento de Fisiología, Facultad de Medicina, Universidad Nacional Autónoma de México, Mexico City, Mexico; ^2^Institute for Biomedical and Biomolecular Sciences, School of Pharmacy and Biomedical Sciences, University of Portsmouth, Portsmouth, United Kingdom; ^3^Department of Anatomy, Charité - Universitätsmedizin Berlin, Berlin, Germany; ^4^I.M. Sechenov First Moscow State Medical University of the Ministry of Healthcare of the Russian Federation (Sechenovskiy University), Moscow, Russia; ^5^Division of Molecular Pharmacology, Department of Pharmacology, Jichi Medical University, Shimotsuke, Japan

**Keywords:** V1b, desmopressin, patch clamp, TASP 0390325, restraint stress

## Abstract

The locus coeruleus (LC) is a brainstem nucleus distinguished by its supply of noradrenaline throughout the central nervous system. Apart from modulating a range of brain functions, such as arousal, cognition and the stress response, LC neuronal excitability also corresponds to the activity of various peripheral systems, such as pelvic viscera and the cardiovascular system. Neurochemically diverse inputs set the tone for LC neuronal activity, which in turn modulates these adaptive physiological and behavioral responses essential for survival. One such LC afferent system which is poorly understood contains the neurohormone arginine-vasopressin (AVP). Here we provide the first demonstration of the molecular and functional characteristics of the LC-AVP system, by characterizing its receptor-specific modulation of identified LC neurons and plasticity in response to stress. High resolution confocal microscopy revealed that immunoreactivity for the AVP receptor 1b (V1b) was located on plasma membranes of noradrenergic and non-noradrenergic LC neurons. In contrast, immunoreactivity for the V1a receptor was exclusively located on LC noradrenergic neurons. No specific signal, either at the mRNA or protein level, was detected for the V2 receptor in the LC. Clusters immunoreactive for V1a-b were located in proximity to profiles immunoreactive for GABAergic and glutamatergic synaptic marker proteins. AVP immunopositive varicosities were also located adjacent to labeling for such synaptic markers. Whole-cell patch clamp electrophysiology revealed that the pharmacological activation of V1b receptors significantly increased the spontaneous activity of 45% (9/20) of recorded noradrenergic neurons, with the remaining 55% (11/20) of cells exhibiting a significant decrease in their basal firing patterns. Blockade of V1a and V1b receptors on their own significantly altered LC neuronal excitability in a similar heterogeneous manner, demonstrating that endogenous AVP sets the basal LC neuronal firing rates. Finally, exposing animals to acute stress increased V1b, but not V1a receptor expression, whilst decreasing AVP immunoreactivity. This study reveals the AVP-V1a-b system as a considerable component of the LC molecular architecture and regulator of LC activity. Since AVP primarily functions as a regulator of homeostasis, the data suggest a novel pathway by modulating the functioning of a brain region that is integral to mediating adaptive responses.

## Introduction

The LC is a cluster of neurons located within the pons distinguished from surrounding cell groups by their production of the neurotransmitter noradrenaline (NA) (Dahlstroem and Fuxe, 1964). Despite the compact size of this nucleus, the principal neurons projects to almost all regions of the brain and spinal cord, thereby serving as the primary source of NA for the central nervous system (Schwarz and Luo, 2015). The result is a LC-NA system that modulates some of the most salient aspects of brain function such as arousal (Carter et al., 2010), attention (Usher et al., 1999), and memory (Sara, 2015). These neural processes are combined to mediate a core responsibility of the LC-NA system, that being the modulation of adaptive responses to emotional and physiological stressors, which is a process essential for survival in an ever dynamic world (Valentino and Van Bockstaele, 2008). This is accomplished by a highly dynamic excitability profile of LC neurons, which results in the release of NA in precise spatiotemporal and brain-state specific patterns. However, this contribution of the LC-NA system to homeostasis extends beyond the CNS. Indeed, changes in the activity of LC neurons have been associated with diverse peripheral physiological states, such as intestinal contractility (Lechner et al., 1997), changes in blood pressure (Curtis et al., 1993), bladder contractility (Rickenbacher et al., 2008) and fluid balance (Godino et al., 2005). Therefore, the factors that govern LC neuronal excitability have the potential to impact on a vast array of physiological processes both within the CNS and in a number of major peripheral organs in health and disease.

The principal noradrenergic neurons of the LC have the ability to fire action potentials spontaneously and independently of synaptic inputs (Williams et al., 1991). However, their firing frequency is strongly modulated by conventional neurotransmitters (Cherubini et al., 1988; Singewald and Philippu, 1998). Often co-expressed with GABAergic and glutamatergic inputs are an assortment of neuropeptides which either exert direct effects on LC neuronal excitability or modulate the effects of the co-released neurotransmitters, and are thus considered as neuromodulators (Zitnik, 2016). In contrast, the role of another LC afferent system, distinguished by the expression of the neurohormone AVP (Rood and De Vries, 2011) is relatively poorly understood in terms of its contribution to LC function.

The primary signature of AVP is that of a hormone that acts on a range of physiological systems and is integral for the maintenance of homeostasis. Primarily produced by the hypothalamus, its multitude of functions include contributing to the regulation of fluid balance (Bankir et al., 2017), blood pressure (Lozic et al., 2018), thermoregulation (Daikoku et al., 2007), stress, (Caldwell et al., 2017) as well as emotional and social behavior (Wu et al., 2015). Although many of these functions mirror those of the LC-NA system detailed above, evidence for the cooperativity of these two homeostatic systems is rather limited. This is due to the dearth of information on the molecular, cellular and behavioral correlates of the AVP system within brain pathways such as the LC.

In the mouse, AVP-immunoreactive axonal profiles have been demonstrated in the region of the pons occupied by the LC (Rood and De Vries, 2011). However, this single labeling study failed to demonstrate the association of such fibers with identified LC neurons or other LC neuromodulatory afferent systems. This is important because the LC is composed of morphologically diverse noradrenergic (Schwarz and Luo, 2015) and neurochemically diverse non-noradrenergic neurons (Corteen et al., 2011). Furthermore, the specific receptors, through which AVP might communicate with LC neurons remain to be identified. Three AVP receptor subtypes have been identified, namely AVP receptor 1a (V1a), 1b (V1b) and 2 (V2) (Thibonnier et al., 2002). Expression of the V1b receptor at the mRNA level has been reported in the pons, but not on identified LC neurons (Young et al., 2006). Evidence for the association of AVP with LC is more developed at the functional level. Recordings from neurons in the LC showed an excitatory effect of AVP in gerbil (Olpe et al., 1987) and rat (Berecek et al., 1987), whilst the injection of AVP into the LC complex, not on identified LC neurons, of cats, altered posture and vestibulospinal reflexes (Andre et al., 1992). However, such effects on LC excitability have yet to be demonstrated in other species, which is an important caveat given the demonstrated species differences of the CNS AVP system (Tribollet et al., 1998). Finally, although the contribution of AVP in the stress response has been demonstrated in specific stress circuits (Herman and Tasker, 2016), the activation of the LC AVP system, during stress, has yet to be demonstrated. Here we provide the first demonstration of the molecular and physiological characteristics of AVP-receptor system within neurochemically defined cellular networks of the mouse LC. We also show that exposing mice to a single episode of stress dramatically alters the expression of the LC AVP-receptor system, providing evidence for the direct interaction of two major homeostatic systems.

## Materials and Methods

All procedures involving animal experiments were approved by the Animal Welfare and Ethical Review Body of the University of Portsmouth and were performed by a personal license holder, under a Home Office-issued project license, in accordance with the Animals (Scientific Procedures) Act, 1986 (United Kingdom) and associated procedures.

### Tissue Preparation for Immunohistochemistry

Adult male C57BL/6J, as well as V1a and V1b receptor specific gene-deleted (Tanoue et al., 2004; Koshimizu et al., 2006) mice, 2 months of age, were used to determine the native AVP and AVP receptor subtype expression patterns, according to a previously published protocol (Corteen et al., 2011). Briefly, under anesthesia, animals were perfused transcardially with 0.9% saline solution for 2 min, followed by 12 min fixation with a fixative consisting of 1% paraformaldehyde and 15% v/v saturated picric acid in 0.1 M phosphate buffer (PB), pH 7.4. The brains were post-fixed over night at room temperature in the same perfusion fixative, sectioned with a vibratome and stored in 0.1 M PB and 0.05% sodium azide until further processing.

### Immunohistochemistry

Non-specific binding of the secondary antibodies was minimized by incubating the sections in TBS-Tx containing 20% normal horse serum (S-2000, Vector Laboratories Inc.) for 2 h. Sections were incubated in a cocktail of primary antibodies overnight at 4°C (Supplementary Table S1). The next day, the sections were washed with TBS-Tx for 30 min after which they were incubated at room temperature in a cocktail of an appropriate mixture of secondary antibodies, conjugated with Alexa Fluor 488, indocarbocyanine (Cy3) and indocarbocyanine (Cy5), all provided by Jackson ImmunoResearch, for 2 h. The sections were washed in TBS-Tx for 30 min after which they were mounted on glass slides, air dried and coverslipped using Vectashield mounting medium (H-1000, Vector Laboratories Inc.).

### Image Acquisition

Sections were examined with a confocal laser-scanning microscope (LSM710 or LSM 880; Zeiss, Oberkochen, Germany) using a Plan Apochromatic 63 x DIC oil objective (NA 1.4, pixel size 0.13 μm). Z-stacks were used for routine evaluation of the labeling. All images presented represent a single optical section. These images were acquired using sequential acquisition of the different channels to avoid cross-talk between fluorophores, with the pinholes adjusted to one Airy unit. Images were processed with the software Zen 2009 Light Edition (Zeiss, Oberkochen, Germany) and exported into Adobe Photoshop. Only brightness and contrast were adjusted for the whole frame, and no part of a frame was enhanced or modified in any way.

### Quantitative Real-Time Polymerase Chain Reaction (qPCR) Detection of V2 mRNA

For complete methods, please see Supplementary Information.

### Whole Cell Patch Clamp Electrophysiology Recordings in Acute Brain Slices of the LC

Recordings were performed in juvenile (postnatal day 25–30) mice according to previously published protocols (Swinny et al., 2010). Briefly, animals were rapidly decapitated and the head placed in ice-cold oxygenated sucrose-cutting solution containing (mM): sucrose (234), KCl (2.5), NaH_2_PO_4_ (1.25), NaHCO_3_ (26), dextrose (10), MgSO_4_ (10), CaCl_2_ (0.5). The brain was rapidly removed and blocked to isolate the brainstem region. The trimmed brain was affixed to a Vibratome equipped with a ceramic blade and submerged in ice cold oxygenated sucrose cutting solution. Horizontal 200 μm slices of the brainstem containing the LC were cut and placed in a holding vial containing extracellular solution (ECS) containing (mM): NaCl (126), KCl (2.95), NaH_2_PO_4_ (1.25), NaHCO_3_ (26), dextrose (10), MgSO_4_ (2), CaCl_2_ (Sigma) for 1 h at 37° C, after which they were kept at room temperature, and transferred one at a time to the recording chamber.

A single slice was placed in the recording chamber and continuously superfused with ECS at 1 ml/min at 32°C. LC neurons were visualized using an Olympus B50 upright microscope fitted with a 40x water-immersion objective, differential interference contrast and infrared filter. Recording pipettes were fashioned from borosilicate glass capillary tubing (1.2 mm o.d., 0.69 mm i.d.; Warner Instruments) using a Narishige PC-10 micropipette puller. Pipettes were filled with potassium gluconate intracellular solution containing: K-Gluconate (70), KCl (70), HEPES (10), EGTA (10), MgCl_2_ (2), CaCl_2_ (1), ATP (2) (Maguire et al., 2014) and 0.1 % biocytin (pH 7.3) to allow *post-hoc* identification of the cell.

A visualized cell was approached with the electrode, a GΩ seal established and the cell membrane ruptured to obtain a whole-cell recording using a Multiclamp 700B amplifier (Molecular Devices, United States). Series resistance was monitored throughout the experiment. If the series resistance of the electrode was unstable or exceeded four times the electrode resistance, electrophysiological data from the cell were discarded. The main criteria for accepting a recording were an action potential amplitude of 65–70 mV, action potential shape characteristic of an LC neuron and membrane potential between -50 and -60 mV. If the cell retained a stable baseline and resistance and did not depolarize over time, the cell was retained for analysis. Signals were digitized by Digidata 1320-series analog-to-digital converter and stored online using pClamp 9 software (Molecular Devices). Only one cell per slice was recorded. The experimental protocol involved recording baseline cell characteristics in current clamp, including FR (Hz), input resistance [derived from the linear portion of a voltage–current plot of hyperpolarizing current steps (MΩ), resting membrane potential (mV), membrane time constant (τ, ms), action potential amplitude (mV) and duration (ms) and AHP amplitude (mV), and AHP *t*_1/2_ duration (measured from the peak of the AHP to half the amplitude of the AHP, in ms)]. After determining baseline characteristics, the drugs were bath-applied for at least 10 min after which the cell characteristics were measured again. Data were analyzed with Clampfit software (Molecular Devices).

Following the recording, the pipette was gently retracted, the slice removed from the recording chamber and submerged in a vial containing 1% paraformaldehyde fixative overnight. Following washing in TBS-Tx buffer, the sections were then incubated with 20% normal horse serum for 1 h at room temperature, followed by incubation with an antibody against TH overnight at 4°C. Following further washing with TBS-Tx, the sections were incubated with a streptavidin conjugated to Alexa Fluor 488 secondary antibody (1:1000) (Molecular Probes, United States), in addition to an appropriate secondary antibody to visualize TH, for 2 h at room temperature. Sections were then imaged on a confocal microscope (Zeiss LSM 710) in order to confirm that the recorded cell was located within the LC and was immunopositive for TH.

### Drugs

All chemicals for the recording solutions were obtained from Sigma. Desmopressin, [d(CH2)51, Tyr(Me)2, Arg8]-Vasopressin and TASP 0390325 were obtained from Tocris, United Kingdom, and dissolved in ECS.

### Acute Restraint Stress

A total of 10 (5 control and 5 stress) male mice, aged 2 months were used in this part of the study. The animals were placed in a rodent Plexiglas restrainer (Harvard Apparatus) for 60 min. The animals were then returned to their home cages for a further 60 min, after which they were killed, using the perfusion-fixation protocol above and the tissue used for quantitative immunohistochemistry.

### Quantification of AVP and V1a-b Receptor Immunoreactivity in the LC of Tissue From Restraint Stress and Control Mice

One hour after the cessation of the stress period, animals were killed by perfusion fixation and the tissue prepared for semi-quantitative analyses of AVP and V1a-b receptor immunoreactivity as above. The quantitative method has been previously described (Gunn et al., 2013) and is detailed in Supplementary Method.

### Statistical Analysis

All quantitative data are presented as the mean ± SEM. Statistical differences between means were assessed using GraphPad Prism software, with the names of statistical tests used indicated in the Results section. A *p*-value less than 0.05 was considered statistically significant.

## Results

The overall aims of the project were to identify the AVP receptor subtypes expressed by LC neurons, the location of these receptors within the cellular networks of the LC, their contribution to LC neuronal excitability and whether they are engaged by acute stress.

### Verification of the Specificity of AVP Receptor Immunoreactivity

**FIGURE 1:**
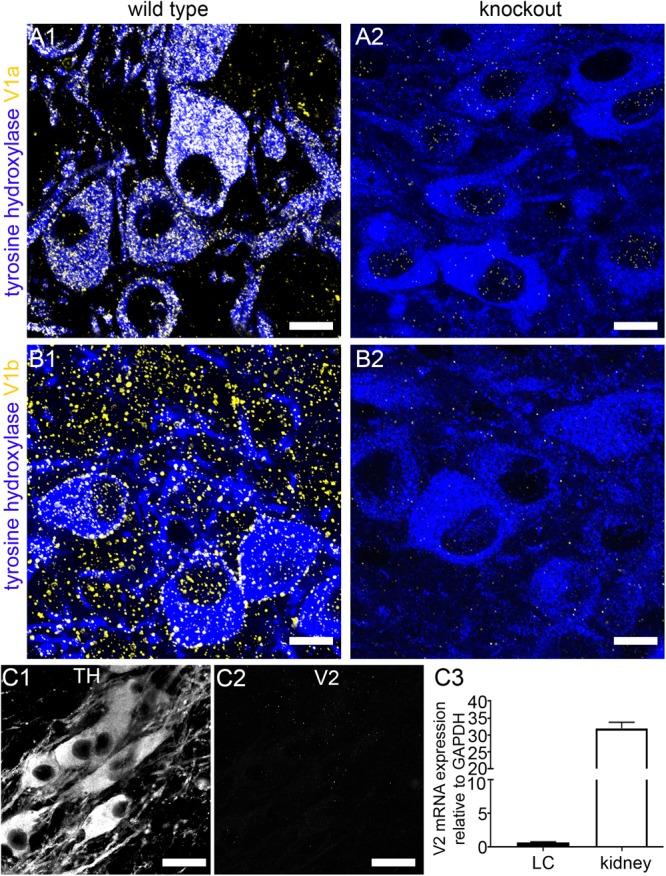
Confirmation of the specificity of the V1a and V1b receptor antibody labeling in the LC. **(A1)** Shows immunoreactivity for tyrosine hydroxylase (TH) (blue) and the V1a receptor (yellow) in tissue from WT mouse. TH is the enzyme essential for noradrenaline synthesis and thus identifies the principal neurons of the LC. V1a signal is exclusively associated with TH-immunopositive profiles. **(A2)** Shows, immunoreactivity for TH (blue) and V1a receptor (yellow) in tissue from a V1a knock out mouse. Only weak, non-specific nuclear signal is evident confirming the specificity of the V1a antibody labeling pattern. **(B1)** Shows immunoreactivity for tyrosine hydroxylase (TH) (blue) and the V1b receptor (yellow) in tissue from WT mouse. V1b signal is associated with TH-immunopositive cells as well as areas not containing TH-immunopositive profiles. **(B2)** Shows, immunoreactivity for TH (blue) and V1b receptor (yellow) in tissue from a V1b knock out mouse. Only weak, non-specific signal is evident confirming the specificity of the V1b antibody labeling pattern. **(C1)** Shows TH immunoreactivity. **(C2)** Shows, in the corresponding field of view, labeling for an antibody against the V2 receptor. No specific signal was detectable. **(C3)** Shows the expression of mRNA for the V2 receptor in tissue from the LC and the kidney which is known to express this receptor subtype. Whilst robust mRNA expression was detected in kidney, negligible expression was evident in LC samples. *N* = 5 animals, with the bars representing the means and the error bars, the SEM. Scale bars **(A,B)**, 10 μm; **(C)** 20 μm.

A key determinant of a direct AVP influence on LC function would be the presence, and subtype, of AVP receptors in this brain region. Since there is evidence for the expression of all AVP receptor subtypes in the brain, from the outset, we sought to identify whether specific AVP receptor subtypes are expressed on identified LC neurons. In tissue from wild type (WT) mice, V1a receptor immunoreactivity presented as individual clusters enriched on the somata and dendrites of LC noradrenergic neurons, identified by immunoreactivity for the NA synthesizing enzyme tyrosine hydroxylase (TH) (Figure 1A1). In tissue from V1a receptor knockout mice, no such specific V1a receptor labeling pattern was detectable, with most of the signal enriched in nuclei or scattered diffusely showing no clear association with any cellular profiles, thus confirming the specificity of the V1a antibody used (Figure 1A2). In WT tissue, immunoreactivity for the V1b receptor also presented as clustered signal on somatodendritic compartments of LC noradrenergic neurons. However, in contrast to V1a, signal for V1b appeared more widespread and was likely contained on LC non-noradrenergic neurons as well (Figure 1B1). This labeling pattern was absent in tissue from V1b receptor knockout mice, thus confirming the specificity of the V1b antibody used (Figure 1B2). No specific labeling for the V2 receptor was detectable in the LC (Figures 1C1,C2). We further verified the lack of V2 receptor expression in the LC, at the mRNA level, by performing quantitative RT-PCR on tissue from the LC and another tissue site known to express this receptor, namely the kidney (*N* = 5 mice). Whilst robust V2 receptor mRNA expression was detected in kidney samples, only negligible amounts were evident in LC samples (Figure 1C3). We therefore conclude that V1a-b receptors are the major subtypes expressed in the mouse LC.

### The V1b Receptor Is Expressed by Noradrenergic and Non-noradrenergic Neurons of the LC in Close Proximity to Excitatory and Inhibitory Synapses

**FIGURE 2:**
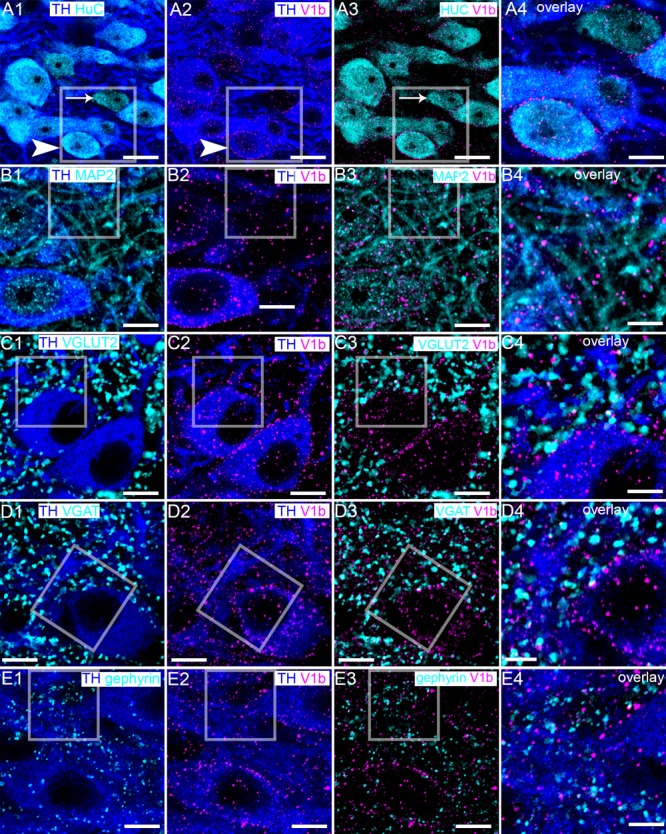
V1b receptor is expressed on noradrenergic and non-noradrenergic LC neurons in close proximity to glutamatergic and GABAergic synapses. **(A1)** Shows immunoreactivity for TH (blue), a marker of noradrenergic neurons and HuC (cyan), a pan-neuronal marker. Thus, in this brain region, HuC identifies noradrenergic (arrowhead) and non-noradrenergic (arrow) neurons of the LC. **(A2)** Shows, in the same field of view, immunoreactivity for TH (blue) and V1b receptor (magenta), confirming its expression in LC noradrenergic neurons (arrowhead). V1b receptor signal presents as individual clusters concentrated on somatodendritic plasma membranes, typifying the expression pattern of a metabotropic receptor. **(A3)** shows, in the same field of view, immunoreactivity for V1b receptor (magenta) and HuC (cyan), confirming V1b receptor expression in LC non-noradrenergic neurons as well (arrow). **(A4)** Is an overlay and magnified view of the boxed area in **(A1–A3)**. **(B1)** Shows immunoreactivity for TH (blue), and microtubule associated protein 2 (MAP2) (cyan), a protein enriched in dendrites. **(B2)** shows, in the same field of view, immunoreactivity for TH (blue) and V1b receptor (magenta), which confirms V1b expression on dendritic compartments. **(B3)** Shows, in the same field of view, the association of immunoreactivity for V1b (magenta) with MAP2 (cyan), confirming V1b expression in dendritic compartments as well. **(B4)** Is an overlay and magnified view of the boxed area in **(B1–B3)**. **(C1)** Shows immunoreactivity for TH (blue), and vesicular glutamate transporter 2 (VGLUT2) (cyan), a protein enriched in glutamatergic axon terminals and used here to identify presynaptic domains of excitatory synapses. **(C2)** Shows, in the same field of view, immunoreactivity for TH (blue) and V1b receptor (magenta). **(C3)** Shows, in the same field of view, the association of immunoreactivity for V1b receptor (magenta) with VGLUT2 (cyan). The close association suggests that the V1b receptor in LC neurons is expressed in close proximity to excitatory synapses. **(C4)** Is an overlay and magnified view of the boxed area in **(C1–C3)**. **(D1)** Shows immunoreactivity for TH (blue), and vesicular GABA transporter (VGAT) (cyan), a protein enriched in GABAergic and glycinergic axon terminals and used here to identify presynaptic domains of inhibitory synapses. **(D2)** Shows, in the same field of view, immunoreactivity for TH (blue) and V1b (magenta). **(D3)** Shows, in the same field of view, the association of immunoreactivity for V1b (magenta) with VGAT (cyan). The close association suggests that V1b on LC neurons is expressed in close proximity to inhibitory synapses as well. **(D4)** Is an overlay and magnified view of the boxed area in **(D1–D3)**. **(E1)** Shows immunoreactivity for TH (blue), and gephyrin (cyan), a protein that anchors GABAA and glycine receptors in inhibitory synapses and used here to identify postsynaptic domains of inhibitory synapses. **(E2)** Shows, in the same field of view, immunoreactivity for TH (blue) and V1b (magenta). **(E3)** Shows, in the same field of view, the association of immunoreactivity for V1b (magenta) with gephyrin (cyan). There is sparse colocalization of clusters immunoreactive for V1b and gephyrin suggesting a limited incorporation of V1b in inhibitory postsynaptic domains, with this receptors located most likely on perisynaptic compartments. **(E4)** Is an overlay and magnified view of the boxed area in **(E1–E3)**. Scale bars **(A1–A3)** 20 μm; **(A4,B–D1–D3)** 10 μm; **(B–D4)** 5 μm.

We first examined the expression profile of the V1b receptor as it appeared to be more widely distributed throughout the LC, compared to the V1a receptor, and is possibly associated with both noradrenergic and non-noradrenergic profiles (compare Figures 1A1,B1). We have shown that the LC is composed of not only noradrenergic neurons, but also a range of neurochemically diverse non-noradrenergic neurons (Figure 2A1) (Corteen et al., 2011). In order to gain an integrative perspective of the role of the AVP system within the cellular networks of the LC, we sought to characterize the location of the V1b receptor throughout LC circuitry. High resolution imaging revealed that V1b receptor immunoreactivity was enriched on the plasma membranes of LC neurons with significantly lower levels of signal located in their cytoplasmic compartments, characteristic of a metabotropic receptor (Figure 2A2). Signal was also located on the somata of non-noradrenergic LC neurons (Figure 2A3), identified by immunoreactivity for the pan-neuronal marker protein HuC (Okano and Darnell, 1997) (Figures 2A3,A4). In addition to somatic labeling, V1b receptor was also located on dendritic profiles, delineated by immunoreactivity for microtubule associated protein 2 (MAP2) (Figure 2B).

This clustered distribution of V1b receptor immunoreactivity across the surfaces of LC neurons overlapped with the locations of previously demonstrated GABAergic (Corteen et al., 2011) and glutamatergic (Seifi et al., 2014) inputs. Furthermore, since AVP has been shown to modulate synaptic transmission (Ostergaard et al., 2014), we investigated the proximity of V1b receptor-immunoreactive clusters to excitatory and inhibitory synaptic marker proteins. Clusters immunoreactive for the vesicular glutamate transporter 2 (VGLUT2) contacted the somatic and dendritic surfaces of TH-immunopositive neurons, thereby identifying glutamate release sites on these neurons (Figure 2C1). Evaluation of immunoreactivity for VGLUT2 alongside the V1b receptor revealed a proportion of closely apposed clusters for either molecule, suggesting that a proportion of V1b receptors function in proximity to glutamatergic synapses (Figure 2C2,C3). However, there was also a notable proportion of V1b receptor-immunoreactive clusters located on cell surfaces which were devoid of VGLUT2-immunoreactive contacts (Figures 2C3,C4). Immunoreactivity for the vesicular GABA transporter (VGAT) was used to identify the domains of inhibitory synaptic inputs. VGAT signal was widely distributed across the somatodendritic surfaces of LC neurons (Figure 2D1). In a similar manner, a proportion of V1b receptor immunoreactive clusters were adjacent to clusters immunoreactive for VGAT, although some, particularly those on somatic membranes were not associated with VGAT signal (Figures 2D2–D4). Since VGAT and VGLUT2 identify presynaptic compartments, we used gephyrin, a protein that anchors GABA_A_ and glycine receptors in the postsynaptic compartments of inhibitory synapses, in order to assess any enrichment of the V1b receptor in postsynaptic junctions. A relatively sparse association of V1b and gephyrin immunoreactivity was evident (Figure 2E). However, at the light microscopical level, it is not possible to determine the precise location of these receptors, or other proteins, in proximity to presynaptic, postsynaptic, perisynaptic or extrasynaptic compartments. Therefore, further ultrastructural analyses, using immunohistochemistry and transmission electron microscopy is required to unequivocally determine the location of these receptors in proximity to synaptic junctions.

### V1b Receptor Activation Has Contrasting Effects on the Spontaneous Firing of LC Noradrenergic Neurons

**FIGURE 3:**
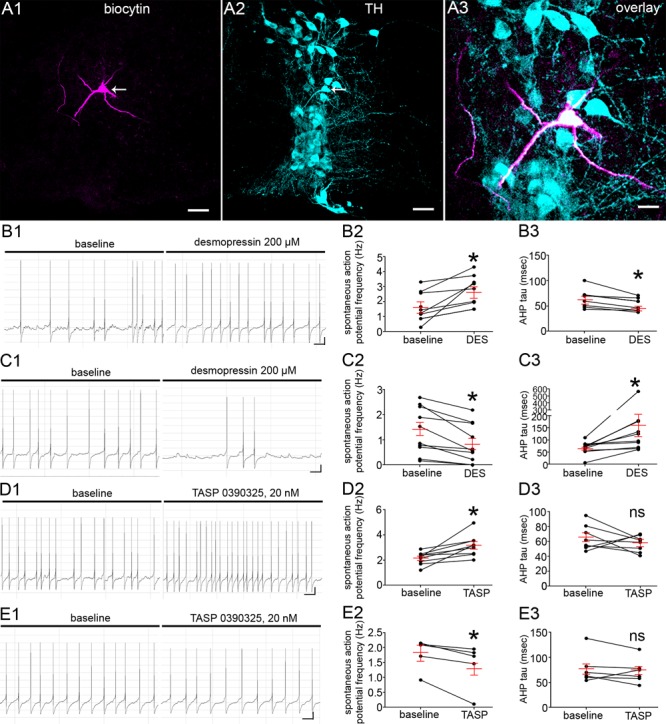
V1b receptor modulation directly alters LC noradrenergic neuronal activity. **(A)** *Post hoc* microscopical confirmation of the molecular identity and location of recorded neurons. **(A1)** Shows immunoreactivity for biocytin which was originally contained within the intracellular solution used for recording the neuron in the whole-cell configuration. Note that immunoreactivity is located exclusively within the single neuron (arrow). **(A2)** Shows immunoreactivity for TH within the same filed of view as **(A1)**, confirming that the recording was from the LC, with the arrow highlighting the recorded cell. **(A3)** Is an overlay and magnified view the cell in **(A1,A2)** confirming that the recorded cell is TH immunopositive and thus a noradrenergic LC neuron. **(B1)** Representative traces of the spontaneous firing pattern of an LC neuron before and after the application of the V1b receptor agonist desmopressin 200 μM, from a cohort of neurons that responded with a significant increase in the frequency of spontaneous action potentials. **(B2)** Quantification of the spontaneous firing rates (Hz) of LC neurons before and after the application of desmopressin (DES). **(B3)** Quantification of the after hyperpolarization time constant (msec) of LC neurons before and after the application of desmopressin. **(C1)** representative traces of the spontaneous firing pattern of an LC neurons before and after the application of the V1b receptor agonist desmopressin 200 μM, from a cohort of neurons that responded with a significant decrease in the frequency of spontaneous action potentials. **(C2)** Quantification of the spontaneous firing rates (Hz) of LC neurons before and after the application of desmopressin. **(C3)** Quantification of the after hyperpolarization time constant (msec) of LC neurons before and after the application of desmopressin. **(D1)** Representative traces of the spontaneous firing pattern of an LC neuron before and after the application of the V1b receptor antagonist TASP 0390325, 20 nM, from a cohort of neurons that responded with a significant increase in the frequency of spontaneous action potentials. **(D2)** Quantification of the spontaneous firing rates (Hz) of LC neurons before and after the application of TASP 039325. **(D3)** Quantification of the afterhyperpolarization time constant (msec) of LC neurons before and after the application of TASP 039325. **(E1)** Representative traces of the spontaneous firing pattern of an LC neuron before and after the application of the V1b antagonist TASP 0390325, 20 nM, from a cohort of neurons that responded with a significant decrease in the frequency of spontaneous action potentials. **(E2)** Quantification of the spontaneous firing rates (Hz) of LC neurons before and after the application of TASP 039325. **(E3)** Quantification of the afterhyperpolarization time constant (msec) of LC neurons before and after the application of TASP 039325. For graphs in **(B–E)**, the dots represent the values for individual cells, the long red bars represents the mean for all cells within that group, and the short red bars represent the SEM. ^∗^*p* < 0.05, paired Student’s *t*-test. Scale bars **(A1,A2)** 50 μm; **(A3)** 20 μm; **(B1,C1,D1,E1)** horizontal bar 1 s, vertical bar 10 mV.

Given the close proximity of V1b receptor signal to synaptic inputs, we next investigated whether the pharmacological activation of V1b receptors alters the spontaneous excitability of identified LC neurons by using whole cell patch clamp electrophysiology in acute brain slices containing the LC. In light of the LC consisting of noradrenergic and non-noradrenergic neurons, all cells were loaded with biocytin during the recording in order to verify their location within the LC and their neurochemical identity. Only cells positively identified as TH-immunopositive and located within the LC core, were included in the analyses (Figure 3A). The spontaneous firing rates (FR) of the recorded neurons were indistinguishable from previously published studies, with the mean ± SEM FR of 1.7 ± 0.1 Hz, ranging from 0.2 Hz to 3.2 Hz, *N* = 50 cells from 15 animals (Supplementary Tables S2–S7). The associated membrane properties of the neurons are also detailed in Supplementary Tables S2–S7. It was noticeable from the outset that V1b receptor activation, by applying the synthetic vasopressin analog desmopressin (200 μM), which acts as an agonist at V1b and V2 receptors, either increased, or decreased the spontaneous FRs of LC neurons. We therefore categorized the recorded neurons into groups which exhibited either an increase or decease in their activity in response to the drug. Desmopressin significantly increased the FR of LC neurons from 1.7 ± 0.3 Hz to 2.9 ± 0.3 Hz, *p* = 0.01, paired Student’s *t*-test; *N* = 9 cells (Figures 3B1,B2). Desmopressin also significantly decreased the afterhyperpolarization (AHP) time constant from 65 ± 6 ms to 53 ± 5 ms, *p* = 0.005, paired Student’s *t*-test; *N* = 8 cells (Figure 3B3). There were no significant differences in any other cell characteristics following the application of desmopressin (Supplementary Table S2). In a separate cohort of cells, recorded under identical conditions, desmopressin significantly decreased the FR of LC neurons from 1.4 ± 0.3 Hz to 0.9 ± 0.2 Hz, *p* = 0.02, paired Student’s *t*-test; *N* = 11 cells (Figures 3C1,C2). In this group of cells, desmopressin also significantly decreased the action potential amplitude (69 ± 1.8 mV to 45.5 ± 9.3 mv, *p* = 0.02, paired Student’s *t*-test; *N* = 11 cells, Figure 3C1) and increased the AHP time constant from 67 ± 8 ms to 167 ± 44 ms, *p* = 0.03, paired Student’s *t*-test; *N* = 11 cells (Figure 3C3). In this group of cells, there were no significant differences in any other cell characteristics following the application of desmopressin (Supplementary Table S2). Thus, the activation of V1b receptors bi-directionally modulates the excitability of LC neurons.

We then explored whether locally released AVP contributes to the basal FR of LC neurons by recording the FR of LC neurons following the blockade of V1b receptors in the presence of the V1b receptor selective antagonist TASP 0390325 (Iijima et al., 2014). In a similar trend to desmopressin, TASP 0390325, 20 nM, either increased (2.2 ± 0.1 Hz to 3.2 ± 0.3 Hz, *p* = 0.01, paired Student’s *t*-test, *N* = 8 cells, Figures 3D1,D2) or decreased (1.7 ± 0.2 Hz to 1.3 ± 0.3 Hz, *p* = 0.03, paired Student’s *t*-test, N = 6 cells, Figures 3E1,E2) the spontaneous FR of neurons. However, in contrast to desmopressin, TASP 0390325 did not significantly alter the AHP time constant either in those cells that showed an increased (65 ± 6 ms to 58 ± 4, *p* = 0.3442, paired Student’s *t*-test; *N* = 8 cells, Figure 3D3) or decreased FR (78 ± 13 ms to 72 ± 10 ms, *p* = 0.3442, paired Student’s *t*-test; *N* = 6 cells, Figure 3E3). This provides evidence that endogenous AVP and V1b receptor activation contributes to the basal level of LC activity.

### The V1a Receptor Is Expressed Exclusively by Noradrenergic Neurons of the LC

**FIGURE 4:**
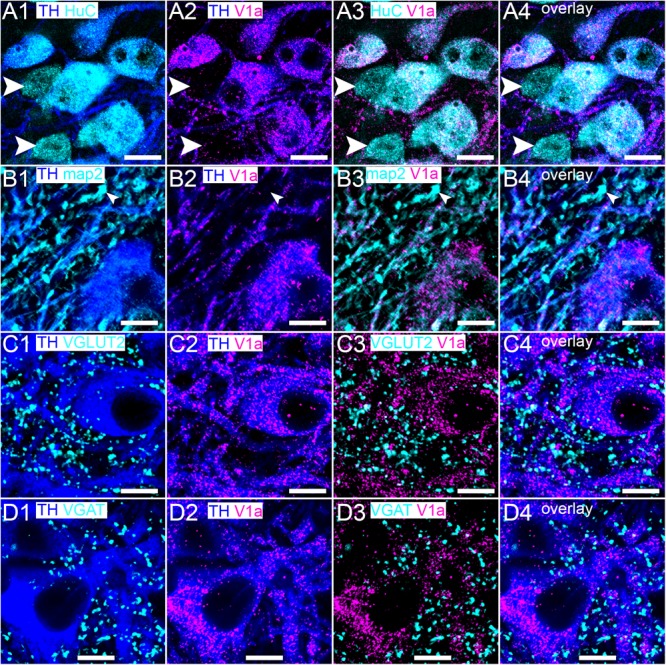
Within the LC, the V1a receptor is expressed exclusively on noradrenergic neurons. **(A1)** Shows immunoreactivity for TH (blue) and HuC (cyan), with the arrowheads highlighting non-noradrenergic neurons of the LC. **(A2)** Shows, in the same field of view, immunoreactivity for TH (blue) and V1a receptor (magenta), confirming that its expression, within the LC, is restricted to noradrenergic neurons and **(A3)** is not located on non-noradrenergic neurons (arrowheads). **(A4)** Is an overlay of **(A1–A3)**. **(B1)** Shows immunoreactivity for TH (blue) and MAP2 (cyan) with the arrowhead highlighting a non-noradrenergic dendritic profile. **(B2)** shows, in the same field of view, immunoreactivity for TH (blue) and V1a receptor (magenta), which confirms V1a expression on noradrenergic profiles and **(B3)** not on non-noradrenergic profiles (arrowhead). **(B4)** Is an overlay of **(B1–B3)**. **(C)** and **(D)** show the association of V1a immunoreactivity with VGLUT2 and VGAT labeling respectively. Scale bars **(A)** 15 μm; **(B–D)** 10 μm.

In contrast to the V1b receptor, V1a receptor immunoreactivity was restricted to LC TH-immunopositive neurons, both on somatic (Figure 4A) and dendritic (Figure 4B) compartments, with TH immunonegative cell, identified by HUC immunoreactivity devoid of any TH signal. In common with the V1b receptor, clusters immunoreactive for V1a were also located, on occasion, in proximity to puncta immunoreactive for VGLUT2 (Figure 4C) and VGAT (Figure 4D).

### V1a Receptor Blockade Has Contrasting Effects on the Spontaneous Firing of LC Noradrenergic Neurons

**FIGURE 5:**
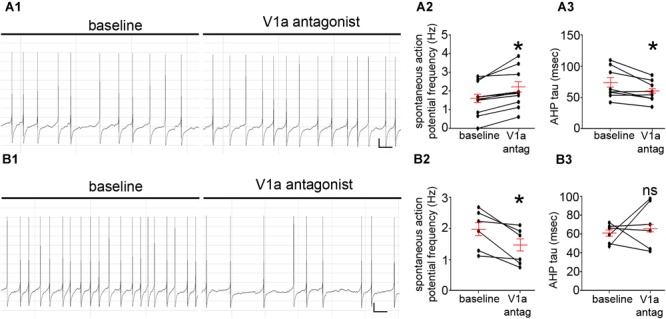
V1a receptor modulation directly alters LC noradrenergic neuronal activity. **(A1)** Representative traces of the spontaneous firing patterns of an LC neuron before and after the application of the V1a receptor antagonist [d(CH2)51, Tyr(Me)2, Arg8]-Vasopressin, 30 nM, from a cohort of neurons that responded with a significant increase in the frequency of spontaneous action potentials. **(A2)** Quantification of the spontaneous firing rates (Hz) of LC neurons before and after the application of the V1a receptor antagonist. **(A3)** Quantification of the afterhyperpolarization time constant (msec) of LC neurons before and after the application of the V1a receptor antagonist. **(B1)** Representative traces of the spontaneous firing patterns of an LC neuron before and after the application of the V1a receptor antagonist, from a cohort of neurons that responded with a significant decrease in the frequency of spontaneous action potentials. **(B2)** Quantification of the spontaneous firing rates (Hz) of LC neurons before and after the application of the V1a receptor antagonist. **(B3)** Quantification of the afterhyperpolarization time constant (msec) of LC neurons before and after the application of the V1a receptor antagonist. In the graphs, the dots represent the values for individual cells, the long red bars represents the mean for all cells within that group, and the short red bars represent the SEM. ^∗^*p* < 0.05, paired Student’s *t*-test. Scale bars **(A1,B1)** horizontal bar 1 s, vertical bar 10 mV.

In a similar trend to the V1b receptor, blockade of the V1a receptor with the antagonist [(d(CH2)51, Tyr(Me)2, Arg8)-Vasopressin, 30 nM] (*n* = 16) also had contrasting effects on LC FR. Indeed, 62% of the cells responded with an increase in their FR (1.6 ± 0.2 Hz to 2.1 ± 0.3 Hz; *p* = 0.0016, paired Student’s *t*-test, *N* = 8 cells, Figures 5A1,A2). However, in contrast to the effects of the V1b antagonist, V1a blockade did result in a significant decrease in AHP of the neurons (73 ± 9 ms to 60 ± 6, *p* = 0.0185, paired Student’s *t*-test; *N* = 8 cells, Figure 5A3). The remaining cells responded with a decrease in their FR (2.0 ± 0.2 Hz to 1.4 ± 0.2 Hz; *p* = 0.0172, paired Student’s *t*-test, *N* = 6 cells, Figures 5B1,B2). However, in these subset of cells, there were no significant differences between the AHP (61 ± 4 ms to 69 ± 10, *p* = 0.5117, paired Student’s *t*-test; *N* = 6 cells, Figure 5B3). This provides evidence that endogenous AVP and V1a-b receptors contributes to the basal level of LC activity. These data are summarized in Supplementary Tables S6, S7.

### AVP-Containing Axons Contact Noradrenergic and Non-noradrenergic Neurons of the LC in Close Proximity to Excitatory and Inhibitory Synapses

The V1a-b antagonist data above provide evidence that AVP within the LC regulates the basal levels of neuronal activity. This could identify a novel pathway that modulates behavioral states associated with changes in both AVP production (Caldwell et al., 2017), and LC neuronal activity (McCall et al., 2015), such as psychosocial stress. For native AVP to exert a significant control over LC-mediated brain functions, there would need to be a considerable distribution of afferents containing this neurohormone throughout the LC. Since previous reports provide only low resolution images of AVP-immunoreactive profiles within the region of the pons occupied by the LC (Rood and De Vries, 2011), we undertook a high resolution characterization of the native association of AVP-containing profiles with identified LC neurons.

**FIGURE 6:**
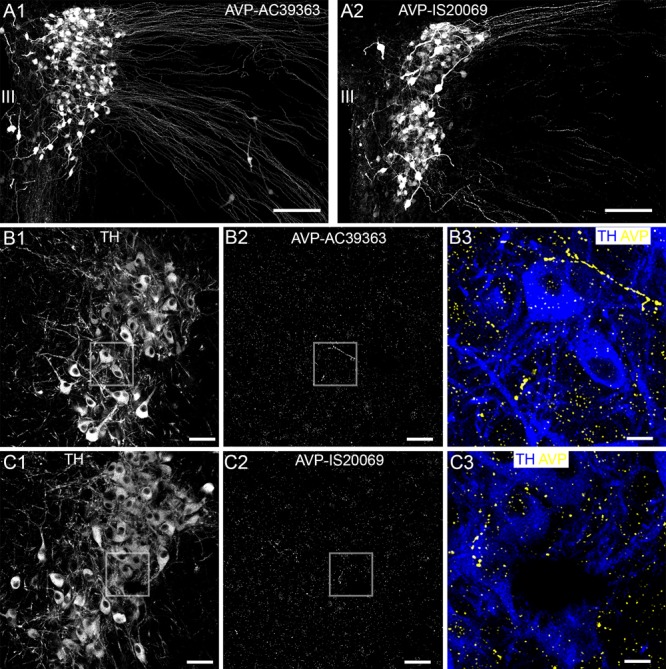
Confirmation of AVP immunoreactivity, in the hypothalamus and LC. **(A1,A2)** Show the comparative immunoreactivity patterns of two different AVP antibodies, in the paraventricular nucleus of the hypothalamus. AC39363 is the antibody obtained from Abcam and IS20069 is the antibody obtained from ImmunoStar, with further details provided in Supplementary Table S1. Both antibodies replicate the characteristic pattern of AVP expression expected in this brain region (Zhang and Hernandez, 2013). **(B,C)** Show the comparative labeling patterns of these AVP antibodies in the LC, when used under conditions identical to those for hypothalamic tissue. **(B3,C3)** Shows that within the LC, AVP immunoreactivity presented as a combination of varicose plexuses and individual clusters contacting somatodendritic surfaces. Scale bars **(A)** 100 μm; **(B1,B2,C1,C2)** 50 μm; **(B3,C3)** 10 μm. III, third ventricle.

The specificity of the LC AVP labeling pattern was replicated using two different AVP antibodies, and the paraventricular nucleus (PVN) as a positive control brain region. Both antibodies provided a pattern of immunoreactivity within the PVN that was consistent with previously published reports (Zhang and Hernandez, 2013) (Figure 6A). However, antibody AC39363 produced a superior signal to noise ratio and was therefore used for remainder of the study. Within the LC, AVP immunoreactivity presented as a mixture of varicose plexuses and individual clusters contacting LC neurons (Figures 6B,C). Importantly, AVP immunoreactivity was distributed through the entire extent of the LC, both within the nuclear core, as well as in the pericoeruleur dendritic region (Figures 6B,C). We found no evidence of specific AVP-immunoreactive neurons within the LC.

**FIGURE 7:**
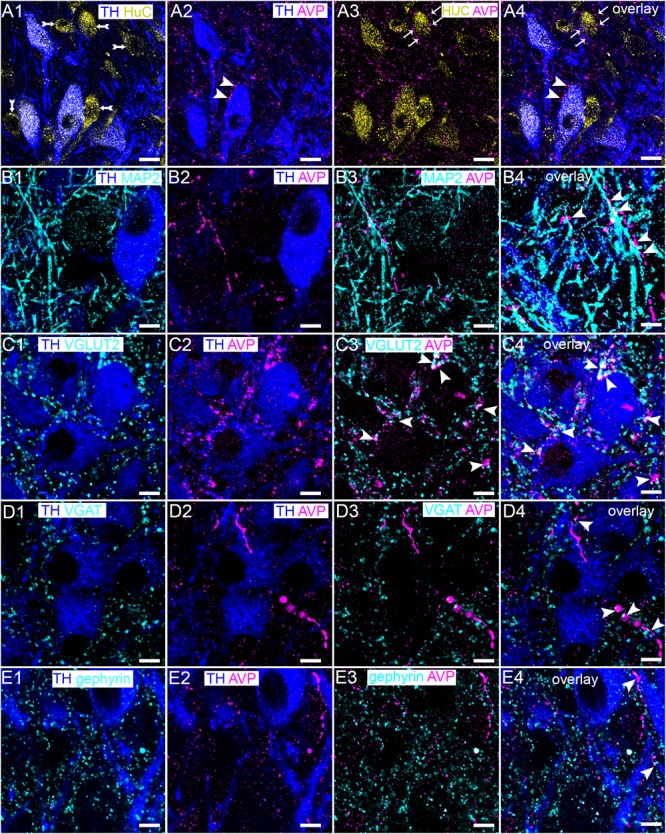
Native AVP-containing axonal profiles contact both noradrenergic and non-noradrenergic LC neurons in close proximity to glutamatergic and GABAergic synapses. **(A1)** Shows immunoreactivity for TH (blue) and HuC (yellow), indicating the distribution of noradrenergic and non-noradrenergic neurons (double arrow) **(A2)** shows, in the same field of view, immunoreactivity for TH (blue) and AVP (magenta) immunoreactive profiles contacting LC noradrenergic neurons (arrowhead). **(A3)** Shows, in the same field of view, immunoreactivity for AVP (magenta) and HuC (yellow), confirming the association of AVP with LC non-noradrenergic neurons as well (arrows). **(A4)** Is an overlay of **(A1–A3)**. **(B1)** Shows immunoreactivity for TH (blue), and MAP2 (cyan). **(B2)** Shows, in the same field of view, immunoreactivity for TH (blue) and AVP (magenta). **(B3)** Shows, in the same field of view, the association of immunoreactivity for AVP (magenta) with MAP2 (cyan). Note the close apposition of the AVP-immunopositive varicosity with the dendritic profile (arrowheads). **(B4)** Is an overlay of **(B1–B3)**. **(C1)** Shows immunoreactivity for TH (blue), and VGLUT2 (cyan). **(C2)** Shows, in the same field of view, immunoreactivity for TH (blue) and AVP (magenta). **(C3)** Shows, in the same field of view, the strong association of immunoreactivity for AVP (magenta) with VGLUT2 (cyan) (arrowheads). **(C4)** Is an overlay of **(C1–C3)**. **(D1)** Shows immunoreactivity for TH (blue), and VGAT (cyan. **(D2)** Shows, in the same field of view, immunoreactivity for TH (blue) and AVP (magenta). **(D3)** Shows, in the same field of view, the association of immunoreactivity for AVP (magenta) with a few VGAT-immunopositive clusters (cyan). **(D4)** Is an overlay of **(D1–D3)**. **(E1)** Shows immunoreactivity for TH (blue), and gephyrin (cyan). **(E2)** Shows, in the same field of view, immunoreactivity for TH (blue) and AVP (magenta). **(E3)** Shows, in the same field of view, the relatively sparse association of immunoreactivity for AVP (magenta) with gephyrin (cyan). **(E4)** Is an overlay of **(E1–E3)**. Scale bars **(A)** 15 μm; **(B–E)** 10 μm.

Upon close inspection, AVP immunoreactive varicosities contacted both noradrenergic (Figure 7A1,A2) and non-noradrenergic (Figure 7A3,A4) neurons of the LC. In addition to this association with somatic compartments, AVP-immunoreactive varicosities also contacted dendritic profiles (Figure 7B), thus closely mirroring the distribution of V1a-b receptors in this brain region. Unfortunately, both antibodies for AVP and V1a-b receptors were raised in the same species, thus precluding co-association studies. Nevertheless, we evaluated the association of AVP immunoreactivity alongside the synaptic marker proteins used for the V1a-b receptors receptor analyses, in an attempt to extrapolate the degree of coherence for their respective locations within these neurons. AVP-immunoreactive profiles were strongly associated with VGLUT2 immunopositive clusters (Figure 7C). However, AVP immunoreactivity showed relatively less association with the inhibitory synaptic markers proteins VGAT (Figure 7D) and gephyrin (Figure 7E). Thus, AVP-immunoreactive axonal profiles are distributed throughout the LC, suggesting a pool of this neurohormone that has the potential to impact on LC neurons.

### AVP-Immunoreactive Profiles Show Sparse Association With Other LC Neuromodulatory Afferent Pathways

**FIGURE 8:**
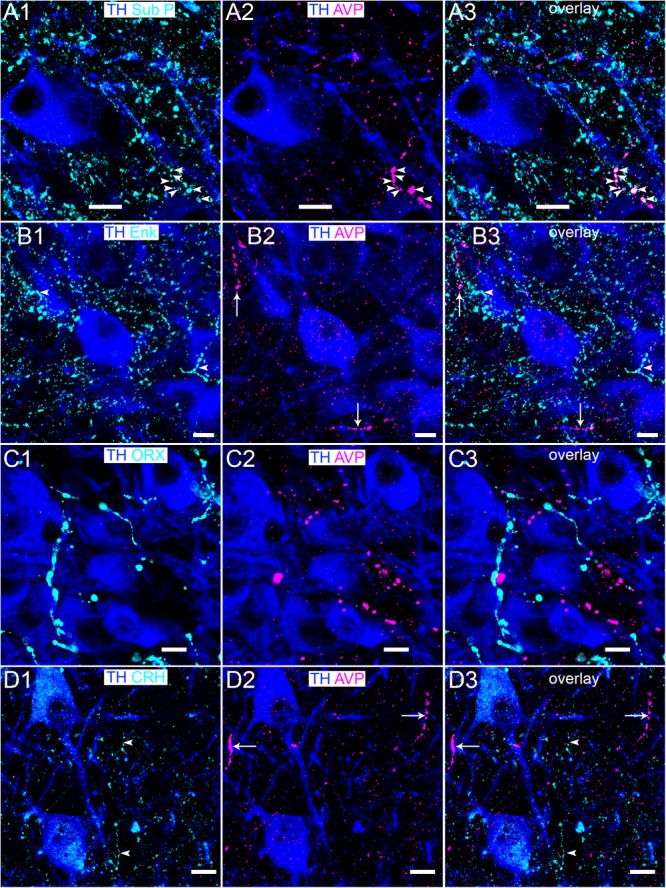
Association of AVP with LC neuromodulatory afferent systems. **(A1)** Immunoreactivity for TH (blue) and substance P (Sub P) (cyan). In the same field of view, **(A2)** immunoreactivity of TH with AVP. **(A3)** Is an overlay of **(A1,A2)** showing isolated instances of colocalization of immunoreactivity for substance P and AVP (arrowheads). Immunoreactivity for TH with **(B1)** enkephalin (ENK), **(C1)** orexin A (ORX) and **(D1)** CRH, with arrowheads highlighting a subset of varicosities in **(B1,D1)**. **(B2,C2,D2)** Show immunoreactivity of TH with AVP in the corresponding fields of view, with AVP immunopositive varicosities highlighted by arrows in **(B2,D2)**. **(B3,C3,D3)** Are overlays of **(B,C,D1,D2)** showing the lack of colocalization of AVP signal with that of the corresponding neuropeptides. Scale bars 10 μm.

It is currently unclear whether LC AVP-immunopositive fibers contain other neuropeptides known to provide modulatory input to this nucleus. We therefore assessed the degree of colocalization between AVP-immunoreactive profiles and varicosities immunopositive for the predominant neuropeptides that have been shown to directly contact LC neurons and alter their excitability, namely substance P (Guyenet and Aghajanian, 1979; Pickel et al., 1979), enkephalin (Valentino and Wehby, 1989; Van Bockstaele et al., 1995), orexin A (Hagan et al., 1999; Horvath et al., 1999), and CRH (Valentino et al., 1983; Van Bockstaele et al., 1996). Only subsets of AVP-immunoreactive varicosities showed colocalization with substance P-immunopositive puncta with the vast majority appearing mutually exclusive (Figure 8A). There was no detectable association between profiles immunoreactive for AVP and enkephalin (Figure 8B), orexin A (Figure 8C), and CRH (Figure 8D). An obvious caveat is that whilst AVP and these other neuropeptides may be contained in the same axons, they may not necessarily be located within the same regions of the axon, thus precluding the confirmation of their co-expression using colocalization analysis. Nevertheless, the data suggests that AVP-containing afferents could represent a distinct axonal pathway within the LC.

### Acute Stress Engages the LC-AVP System

A key contribution of the LC to brain function is the modulation of behavior that allows for adaptation to psychosocial stress (Valentino and Van Bockstaele, 2008). The neurohormone corticotrophin releasing hormone (CRH) (Vale et al., 1981), released from afferents originating from the amygdala (Van Bockstaele et al., 2001), is the primary driver of the increased activity of LC neurons that underlies this behavioral response (Valentino et al., 1983; McCall et al., 2015). However, AVP is also released, together with CRH, from the hypothalamus, as part of the hypothalamic-pituitary-adrenal stress axis (O’connor et al., 2000). Given the evidence that both AVP and CRH are contained in afferents that target LC neurons and both have now been shown to alter LC neuronal activity (this study for AVP), we explored whether acute stress engages the LC-AVP system following exposure to 1 h of restraint stress (Buynitsky and Mostofsky, 2009).

**FIGURE 9:**
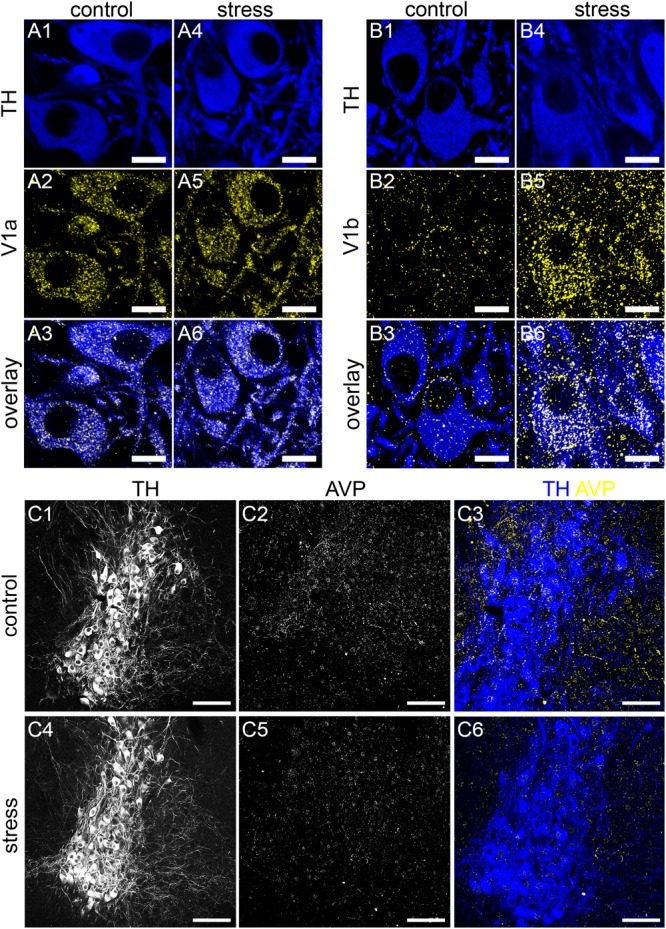
Acute stress engages the AVP-V1a-b system in the LC. **(A1)** Shows TH immunoreactivity in the LC from a control mouse. **(A2)** Shows, in the same field of view, immunoreactivity for the V1a receptor. **(A3)** Is an overlay of **(A1,A2)**. **(A4)** Shows TH immunoreactivity in the LC from tissue of a mouse exposed to 1 h of restraint stress and killed an hour later, processed and imaged under conditions identical to control subjects. **(A5)** Shows, in the same field of view, immunoreactivity for V1a, with no discernible difference in labeling pattern and intensity evident between stress and control samples. **(A6)** Is an overlay of **(A4,A5)**. **(B1)** Shows TH immunoreactivity in the LC from a control mouse. **(B2)** Shows, in the same field of view, immunoreactivity for the V1b receptor. **(B3)** Is an overlay of **(B1,B2)**. **(B4)** Shows TH immunoreactivity in the LC from tissue of a mouse exposed to 1 h of restraint stress. **(B5)** Shows, the same field of view, immunoreactivity for V1b. A dramatic stress-induced increase in the intensity of V1b signal is noticeable, both on cell surfaces but also in cytoplasmic components, suggesting a stress-induced internalization, presumably via prolonged activation. **(B6)** Is an overlay of **(B4,B5)**, which exemplifies the differences between the immunoreactivity patterns in control and stress samples. **(C1)** Shows TH immunoreactivity in the LC from a control mouse. **(C2)** Shows, the same field of view, immunoreactivity for AVP. **(C3)** Is an overlay of **(C1,C2)** with TH and AVP pseudo-colored blue and yellow respectively. **(C4)** Shows TH immunoreactivity in the LC from tissue of a mouse exposed to 1 h of restraint stress and killed an hour later, processed and imaged under conditions to identical control subjects. **(C5)** Shows, the same field of view, immunoreactivity for AVP. A stress-induced decrease in the intensity of AVP signal is noticeable as well as the relative lack of immunoreactive varicosities. This is exemplified in **(C6)**, when compared to **(C3)**. Scale bars **(A,B)** 10 μm; **(C)** 100 μm.

The intensity and pattern of V1a receptor immunoreactivity was indistinguishable in tissue from control and stress animals, processed and imaged under identical conditions (Figure 9A). Semi-quantitative analysis of the density (number of clusters per area) of V1a receptor immunoreactive clusters revealed no significant differences between control and stress subjects (control: 5782 ± 409 clusters per 100 μm^2^ versus stress: 4844 ± 753 clusters per 100 μm^2^; *p* = 0.3054, unpaired Student’s *t*-test, *N* = 5 animals).

In contrast, we detected a significant increase in the number of V1b receptor immunoreactive clusters, especially within cytoplasmic domains (Figure 9B). Semi-quantitative analysis of the density (number of clusters per area) of V1b receptor immunoreactive clusters revealed a significant increase in tissue in stress subjects (control: 945 ± 173 clusters per 100 μm^2^ versus stress: 4462 ± 307 clusters per 100 μm^2^; *p* < 0.0001 unpaired Student’s *t*-test, N = 5 animals). This dramatic stress-induced alteration in V1b receptor immunoreactivity was accompanied by a change in the AVP immunoreactivity pattern. In tissue from control animals, AVP immunoreactivity presented as a mixture of varicose plexuses and individual clusters throughout the LC (Figures 9C1–C3). However, in tissue from stress subjects, processed and imaged under conditions identical to control, there was a noticeable decrease in the intensity of AVP immunoreactivity within the LC, with a relative lack of varicose plexuses (Figures 9C4–C6). Because AVP signal presented as a mixture of varicose plexuses and individual clusters, we performed the semi-quantitative analysis using fluorescence intensity which will incorporate both patterns of signal. Stress induced a moderate, though statistically significant decrease in the AVP fluorescence intensity [control: 35 ± 2 arbitrary units (AU) versus stress: 27 ± 2 AU, *p* = 0.04, unpaired Student’s *t*-test, *N* = 5 animals]. This suggests that stress engages the LC AVP-receptor system.

## Discussion

The current study provides the first demonstration of the association of AVP-immunopositive axons with identified noradrenergic and non-noradrenergic neurons of the LC. These AVP-containing axons showed only a sparse association with another neuropeptide afferent system within this brain region, namely substance P. This suggests that AVP input to the LC represents a pathway distinct to the myriad of neuropeptide systems that modulate information transfer to this nucleus. Furthermore, we identify the specific AVP receptors within the LC, by localizing the V1a and V1b receptors to the somatodendritic surfaces of these different LC cell types. The modulation of both receptor subtypes induced heterogeneity in the excitability of LC noradrenergic neurons, manifesting as either an increase or decrease in their spontaneous FR. Given the strong association between LC FR and the level of NA release, this suggests that this individual neurohormone is capable of both increasing and diminishing CNS NA levels. Finally, we provide evidence that exposing mice to a form of stress which generally induces adaptive behavioral and physiological responses, results in molecular plasticity in the AVP-receptor system. This potentially identifies AVP as an integral component of the molecular machinery utilized in LC-mediated homeostatic responses.

One of the most intriguing findings of the study is that V1a-b receptor ligands had contrasting effects on the spontaneous firing activity of LC neurons. *Post hoc* neurochemical analyses confirmed that all recorded neurons were located within the LC and were noradrenergic. There could be a number of reasons why, presumably, the activation of a single receptor type, on a specific cell type, induces contrasting effects on cellular excitability. Firstly, contrasting downstream receptor signaling cascades could be induced in different populations of LC noradrenergic neurons, following their activation. As receptors coupled to G protein (GPCRs), V1a and V1b receptors conventionally couple to the Gq and phospholipase C (PLC) pathway with the V2 receptor engaging the Gs and adenylate cyclase (Gs-cAMP) pathway (Koshimizu et al., 2012). However, individual AVP receptors engaging differing secondary messenger cascades is not unprecedented. Indeed, multiple mechanisms have been identified which result in the differential activation of secondary messenger pathways by individual GPCRs. These include receptors adopting different active and inactive conformational states when bound to ligands (Kenakin, 2003), having the ability to homo- or heterodimerise, leading to variations in their pharmacological properties (Park and Palczewski, 2005) and the location of receptors within specialized microdomains of the plasma membrane such as rafts or caveolae (Guzzi et al., 2002). Such versatility in signal transduction has been exemplified by the V1b receptor which has been shown to activate either the IP or cAMP pathways (Jard et al., 1986; Thibonnier et al., 1997; Sabatier et al., 1998). Furthermore, the V1b receptor is capable of simultaneously activating both the Gq/11-inositol phosphate (IP) and Gs-cAMP pathways (Orcel et al., 2009). We are unable to distinguish from our data which of the above variables could contribute to the differences in the effects of desmopressin on the recorded neurons. A fundamental question is whether the directionality of the AVP response depends on the activity or metabolic state of the neuron, or it is a central feature of specific sub-populations of LC neurons. If the former, this could identify a role for AVP driving LC activity toward basal levels following various triggers which elevate excitability, such as stress (discussed below), or those which decrease LC activity, such the release of opioids, thus in keeping with its universal role of a homeostatic regulator. If the latter applies, this could be a mechanism for magnifying the diversity of LC noradrenergic neuron sub-populations. Indeed, the LC has been shown to be composed of genetically diverse noradrenergic neurons which are considered to represent different cell types (Robertson et al., 2013; Plummer et al., 2017). Future high resolution molecular, physiological and behavioral correlative studies are required to address these questions. Irrespective, and given the correlation between LC FR and NA release, these data identify the AVP-V1a-b receptor system as possibly being capable of both enhancing or diminishing NA release within the brain.

Precisely how AVP receptors change the spontaneous FR of LC neurons is unclear. This could be an indirect response to the modulation of local neurotransmitters, given the close association of these receptors with GABA and glutamate synaptic molecular machinery demonstrated in this study, and functional evidence in other brain regions (Raggenbass, 2008; Rood and Beck, 2014). Although we recorded from only noradrenergic neurons of the LC, our immunohistochemical data confirm the presence of V1b receptors in non-noradrenergic LC neurons. These neurons bear the neurochemical hallmarks of inhibitory interneurons (Aston-Jones et al., 2004; Corteen et al., 2011). If so, applied desmopressin could induce the release of their neurotransmitter content locally, thereby impacting on the excitability of the recorded noradrenergic neuron. Paired recordings between identified interneuron-principal cell pairs would be essential to understand this dynamic of the AVP-LC system.

However, based on the changes in membrane properties induced by desmopressin, AVP could also directly alter LC neuronal excitability. In addition to changes in spontaneous action potential frequency, desmopressin also altered the membrane time constant for the AHP, thus impacting on the time taken for the neuron to repolarise and thus be capable of initiating the next action potential. This effect on the LC inter-spike interval, by other neurohormones such as CRH, has been shown to be modulated by potassium conductances (Jedema and Grace, 2004). Furthermore, CRH, in different cell types, has been shown to engage different secondary messenger pathways (Grammatopoulos et al., 2001), resulting in either the activation or inhibition of specific ion channels (Aldenhoff et al., 1983; Rainnie et al., 1992; Yu and Shinnick-Gallagher, 1998). Finally, desmopressin also significantly altered the action potential amplitude, but only in those cells which exhibited a decreased FR. It is therefore reasonable to speculate that AVP directly alters LC activity via the downstream modulation of ion channels associated with spontaneous FR. However, desmopressin did not alter the input resistance of the entire cell, thus arguing against any potential impact on membrane channel gating. Future studies investigating LC neuronal excitability in AVP receptor subtype-specific knock out mice will hopefully confirm the receptor subtype signaling cascaded in individual LC neurons.

A novel discovery was the changes in the patterns of AVP and V1b receptor immunoreactivity following exposure to acute stress. Importantly, V1a receptor immunoreactivity remained unchanged. An indicator of metabotropic receptor activation is receptor internalization from plasma membranes and incorporation within cytoplasmic compartments. Thus, the dramatic stress-induced internalization of V1b localization suggests engagement of stress signals with this receptor system and a potential pathway mediating the LC stress response. Future functional and behavioral studies are essential to identify the underlying mechanisms. We deliberately chose an acute stress protocol that is likely to engage adaptive or homeostatic pathways throughout the body including the LC. This has been widely demonstrated with the acute exposure to restraint stress used in this study (Buynitsky and Mostofsky, 2009). LC neurons are exquisitely sensitive to both emotional and physiological stressors, responding with an increased FR (Valentino and Van Bockstaele, 2008). The principal mediator of this stress-induced LC excitability is CRH, originating from a variety of brain centers (Valentino et al., 1992). Although AVP release from the hypothalamus coincides with that of CRH and the stress response (Kormos and Gaszner, 2013), its involvement in the LC stress pathways has yet to be documented. However, this would not be surprising, given the overlap of the LC, and AVP in general, in mediating adaptive or homeostatic responses to various psychological and physiological challenges (Valentino and Van Bockstaele, 2008; Koshimizu et al., 2012). If so, this could signal a novel partnership with CRH in regulating LC activity during times of stress. Precisely how these systems might cooperate is unclear. An important distinction would be their contribution to resting LC FR. CRH antagonists do not alter resting LC FR thus indicating that this aspect of LC physiology is not mediated by the CRH system. In contrast, this study demonstrated that selective V1a-b receptor antagonists, on their own, significantly altered basal LC FR, indicating that endogenous AVP sets the tone of LC activity. Another difference would be the ability of the AVP-V1a-b receptor system to bi-directionally modulate LC activity. This distinguishes AVP-V1a-b system not only from the CRH but other neuropeptide systems that influence LC activity by either selectively increasing or decreasing activity, but never both (Zitnik, 2016). It would be intriguing to explore whether AVP would increase, or decrease, the activity of LC neurons, in a behavioral or physiological state-dependent manner. CRH is called upon during the stress response to elevate LC activity and enhance NA release. However, central to an adaptive or healthy stress system is the appropriate cessation of the stress response once the organism has effectively contended with the challenge. If AVP did work in tandem with CRH in this process, it could provide a counter measure by reversing any increase in LC activity, thereby ensuring effective resetting of basal LC activity. This could provide unique opportunities for V1a-b receptor ligands in psychiatric disorders associated with an impaired stress response and LC dysregulation, such as anxiety and depression (Itoi and Sugimoto, 2010). Indeed, in humans, the dysregulation of brain and CSF AVP levels are associated with a number of anxiety disorders (Neumann and Landgraf, 2012). Furthermore, the pharmacological blockade of either the V1a or V1b receptors showed anxiolytic and antidepressant actions (Koshimizu et al., 2012; Caldwell et al., 2017). The actions of these receptors on such emotional states have been attributed to changes in the functions of a range of brain regions such as the hypothalamus, the bed nucleus of the stria terminallis and amygdala, with the LC largely ignored. The current data suggest that the LC should be considered as a site of action for such agents in altering such psychiatric symptoms. The use of cell type-specific AVP receptor knock out mice will be instrumental in dissecting the association of specific brain regions and AVP receptor subtypes in these brain functions.

In summary, the study provides a new vista on the central AVP system, by providing the molecular and physiological constructs of this neurohormone, and its associated receptor system, in the mouse LC. The data will serve as the basis to further dissect the contribution of what is conventionally a homeostatic system, to the functioning of a brain region that is integral to mediating adaptive responses to a variety of threats.

## Author Contributions

JS conceived and led the study. EC-L, LK, MS, and JS designed the experiments. EC-L, LK, MS, TJ, VH, LZ, and JS performed the experiments and analyzed the data. VH, TG, KM, TK, and LZ contributed unique resources. JS wrote the manuscript with all authors approving the final version.

## Conflict of Interest Statement

The authors declare that the research was conducted in the absence of any commercial or financial relationships that could be construed as a potential conflict of interest.
