# Supplementary material for: Dynamic Modulation of Mouse Locus Coeruleus Neurons by Vasopressin 1a and 1b Receptors
Source: Front Neurosci. 2018 Dec 10;12:919. doi: 10.3389/fnins.2018.00919 (PMC6295453; doi:10.3389/fnins.2018.00919)
Supplement: Supplementary file 1 [file Data_Sheet_1.PDF]

## Supplementary Information

## Methods

*Quantification of AVP and V1b receptor immunoreactivity in the LC of tissue from restraint stress and control mice*

One hour after the cessation of the stress period, animals were killed by perfusion fixation and the tissue prepared for semi-quantitative analyses of AVP and V1b receptor immunoreactivity as above. The quantitative method has been previously described (Gunn et al., 2013) and is detailed in SI Methods. V1b immunoreactivity presented as distinct, individual puncta. We therefore were able to quantify the density of expression, expressed as the number of individual puncta per 100 $\mu m^2$. In contrast to the individual or discrete punctate immunoreactivity pattern of V1b, AVP immunoreactivity pattern within the LC presented as a combination of clusters and axonal varicosities. Therefore, estimate the changes in the levels of both immunoreactivity profiles for AVP, we adopted a densitometry approach by quantifying total fluorescence intensity. The imaging and quantification was performed as follows: within a tissue section, 3 fields of view (FOVs) were selected within the LC core. Z-stacks consisting of 3 optical sections spaced 5 µm apart in the Z-plane were acquired for each FOV. The dimensions of each optical section were 85 µm × 85 µm × 1 µm in the X-Y-Z planes. In ImageJ, thresholding of the images was performed using images obtained from in tissue sections reacted with only the secondary antibodies, with the background fluorescence then subtracted from the values obtained for individual markers. Within an optical section, the density (number of 1b clusters per unit area) was quantified by using the cluster analysis feature of ImageJ. For AVP, mean fluorescence intensity feature of ImageJ was used for quantification. A value for each FOV was obtained by computing the average from the optical sections contained within a FOV. The means ± SD for all FOV between sections and between either control or ELS animals was compared for statistical differences using Kruskal–Wallis one-way ANOVA. These values were then pooled because there were no statistical differences ($p > 0.05$) between the values for FOV between sections and between animals for a particular treatment. The average value from all FOVs and sections per animal were computed for individual animals. This average value for an individual animal was then considered an N of 1. All sections were processed and imaged under identical conditions and analyses were performed blind.

*Quantitative Real-Time Polymerase Chain Reaction (qPCR)*

Adult male mice (N = 5) were killed by cervical dislocation and tissue homogenates of the LC and kidney prepared. RNA was extracted from the samples using an RNeasy mini kit (Qiagen, 74104) according to the manufacturer's protocol. Equal amount of RNA from each tissue was reverse-transcribed into first-stand cDNA in the following reaction: 2 µl of reverse transcription buffer (BioLabs), 1 µl of oligo(dT)s (ThermoFisher Scientific), 1µ DNTPs (ThermoFisher Scientific), 0.5 µl of M-Mulv reverse transcriptase (Applied Biosystems) and 0.5 µl of RiboLock RNAse Inhibitor (ThermoFisher Scientific). Quantitative PCR (qPCR) amplification was performed in 96-well plates in a mastermix for probes (Roche, Burgess Hill, UK) and run on a LightCycler® 96 System (Roche). The qPCR amplifications for the mouse *V2* (assay ID: Mm00517071_m1) gene was performed using pre-designed Taqman primers/probes purchased from Life Technologies (ThermoFisher scientific). *Gapdh* (assay ID: Mm99999915_g1) gene expression was used as the housekeeping gene in various reactions. The qPCR cycling conditions entailed 95°C for 10 mins and 40 cycles of 95°C for 15 sec and 60°C for 60 seconds (LightCycler® 96 System, Roche). Standard curves were generated for each gene using serial dilutions of a known amount of mRNA extracted from each organ which

were then reverse transcribed into cDNA. Each measurement was performed in duplicate and each Ct value was then converted into ng mRNA using linear regression analysis of the standard curve (Microsoft Excel). Each ng mRNA value was then normalised against the ng housekeeping gene level within the same sample and the mean mRNA levels for every sample was finally calculated and compared across tissue samples.

## Supplementary Information Tables

Table 1
Details of primary antibodies used in the study

| Antibody | Host | Dilution | Source, cat number | Specificity/Reference |
|---|---|---|---|---|
| AVP | rabbit | 1:3000 | Abcam, ab39363 | Signal could be eliminated by co-incubation with AVP, (Kerman et al., 2006) |
| AVP | rabbit | 1:2000 | Immunostar, 20069 | Preadsorption with synthetic vasopressin peptide (10 µM) resulted in a complete loss of immunolabeling in the paraventricular nucleus of rodent hypothalamus, (Villanueva et al., 2012) |
| AVP receptor 1a | rabbit | 1:4000 | Kerim Mutig and Torsten Giesecke Institute of Vegetative Anatomy, Charité – Universitätsmedizin Berlin, Germany | No specific signal in knockout mouse, this study. |
| AVP receptor 1b | rabbit | 1:5000 | Kerim Mutig and Torsten Giesecke Institute of Vegetative Anatomy, Charité – Universitätsmedizin Berlin, Germany | No specific signal in knockout mouse, this study. |
| AVP receptor 2 | rabbit | 1:250 | Millipore, AB1797P | (Sato et al., 2011) |
| CRH | guinea pig | 1:10000 | Peninsula Laboratories, T-5007.0050 | Expression patterns as shown in previous studies |
| Enkephalin | mouse | 1:3000 | Abcam, ab150346 | (Cuello et al., 1984) |
| Gephyrin | mouse | 1:1000 | Synaptic systems, 147 021 | Knockout mouse, (Pfeiffer et al., 1984) |
| HuC | mouse | 1:500 | ThermoFisher, A-21271 | Expression patterns as shown in previous studies |
| MAP2 | chicken | 1:500 | Aves Labs, MAP0607 | Expression patterns as shown in previous studies |
| Orexin-A | mouse | 1:4000 | Abcam, ab89886 | Expression patterns as shown in previous studies |
| Substance P | guinea pig | 1:500 | LSBio, LS-C76141 | Expression patterns as shown in previous studies |
| Tyrosine hydroxylase | sheep | 1:3000 | Abcam, ab113 | Expression patterns as shown in previous studies |
| VGAT | guinea pig | 1:3000 | FRONTIER INSTITUTE, VGAT-GP-Af1000 | Preadsorption with peptide resulted in a complete loss of immunolabeling,(Miyazaki et al., 2003) |
| VGLUT2 | guinea pig | 1:3000 | FRONTIER INSTITUTE, VGluT2-GP-Af810 | Preadsorption with peptide resulted in a complete loss of immunolabeling, (Miyazaki et al., 2003) |

Table 2
Summary of basic membrane characteristics of noradrenergic LC neurons that responded with an increase in their spontaneous firing rate following the application of the V1b agonist desmopressin. Data are presented as the mean ± SEM.

| Parameter (N) = number of cells | Baseline | Desmopressin | *P* (paired Student's *t* test) |
|---|---|---|---|
| Spontaneous firing rate, Hz (9) | 1.7 ± 0.3 | 2.8 ± 0.3 | 0.01 |
| Resting membrane potential, mV (9) | - 49.7 ± 0.9 | - 50.2 ± 2.3 | 0.83 |
| Threshold for action potential, mV (9) | -36.0± 1.7 | -37.0 ± 2.8 | 0.72 |
| Amplitude of action potential, mV (9) | 68.0 ± 1.2 | 64.6 ± 3.0 | 0.20 |
| Cells spontaneously firing action potentials, % (9) | 100 | 100 | - |
| Input resistance, MΩ (9) | 0.30 ± 0.02 | 0.31 ± 0.01 | 0.71 |
| Membrane time constant, ms (9) | 37.6 ± 4.6 | 41.7 ± 3.7 | 0.10 |
| AHP time constant, ms (8) | 65 ± 6 | 53 ± 5 | 0.005 |

Table 3
Summary of basic membrane characteristics of noradrenergic LC neurons that responded with a decrease in their spontaneous firing rate following the application of the V1b agonist desmopressin. Data are presented as the mean ± SEM.

| Parameter (N) = number of cells | Baseline | Desmopressin | *P* (paired Student's *t* test) |
|---|---|---|---|
| Spontaneous firing rate, Hz (11) | 1.3 ± 0.3 | 0.9 ± 0.2 | 0.02 |
| Resting membrane potential, mV (11) | -50.4 ± 0.6 | -52.0 ± 0.7 | 0.12 |
| Threshold for action potential, mV (11) | -35.3 ± 0.8 | -26.9 ± 5.3 | 0.10 |
| Amplitude of action potential, mV (11) | 69.0 ± 1.8 | 45.5 ± 9.3 | 0.02 |
| Cells spontaneously firing action potentials, % (11) | 100 | 82 | - |
| Input resistance, MΩ (11) | 0.36 ± 0.02 | 0.37 ± 0.04 | 0.79 |
| Membrane time constant, ms (11) | 42.1 ± 1.9 | 45.1 ± 3.2 | 0.46 |
| AHP time constant, ms (11) | 67 ± 8 | 167 ± 44 | 0.03 |

Table 4

Summary of basic membrane characteristics of noradrenergic LC neurons that responded with an increase their spontaneous firing rate following the application of the V1b antagonist TASP 0390325, 20 nM. Data are presented as the mean ± SEM.

| Parameter (N) = number of cells | Baseline | TASP 0390325, 20 nM | *P* (paired Student's *t* test) |
|---|---|---|---|
| Spontaneous firing rate, Hz (8) | 2.2 ± 0.1 | 3.2 ± 0.3 | 0.01 |
| Resting membrane potential, mV (8) | -49.6 ± 1.0 | -49.6 ± 1.2 | 0.87 |
| Threshold for action potential, mV (8) | -35.2 ± 1.1 | -36.2 ± 1.0 | 0.06 |
| Amplitude of action potential, mV (8) | 60.9 ± 3.1 | 58.8 ± 1.0 | 0.63 |
| Cells spontaneously firing action potentials, % (8) | 100 | 100 | - |
| Input resistance, MΩ (8) | 0.334 ± 0.03 | 0.33 ± 0.03 | 0.43 |
| Membrane time constant, ms (8) | 53.1 ± 10.5 | 42 ± 3.8 | 0.88 |
| AHP time constant, ms | 65 ± 6 | 58 ± 4 | 0.34 |

Table 5

Summary of basic membrane characteristics of noradrenergic LC neurons that responded with a decrease in their spontaneous firing rate following the application of the V1b antagonist TASP 0390325, 20 nM. Data are presented as the mean ± SEM.

| Parameter (N) = number of cells | Baseline | TASP 0390325, 20 nM | *P* (paired Student's *t* test) |
|---|---|---|---|
| Spontaneous firing rate, Hz (6) | 1.7 ± 0.2 | 1.34 ± 0.23 | 0.03 |
| Resting membrane potential, mV (6) | -50.3 ± 0.8 | -51.4 ± 0.8 | 0.10 |
| Threshold for action potential, mV (6) | -34.2 ± 0.6 | -35.1 ± 0.6 | 0.07 |
| Amplitude of action potential, mV (6) | 67.2 ± 1.4 | 60.9 ± 3.0 | 0.08 |
| Cells spontaneously firing action potentials, % (6) | 100 | 100 | - |
| Input resistance, MΩ (6) | 0.34 ± 0.03 | 0.33 ± 0.03 | 0.39 |
| Membrane time constant, ms (6) | 53.1 ± 10.5 | 48.1 ± 5.1 | 0.35 |
| AHP time constant, ms | 78 ± 13 | 72 ± 10 | 0.40 |

Table 6

Summary of basic membrane characteristics of noradrenergic LC neurons that responded with an increase in their spontaneous firing rate following the application of the V1a antagonist ((d(CH2)51, Tyr(Me)2, Arg8)-Vasopressin, 30 nM. Data are presented as the mean ± SEM.

| Parameter<br>(N) = number of cells | Baseline | V1a antagonist | *P* (paired Student's *t* test) |
|---|---|---|---|
| Spontaneous firing rate, Hz (10) | 1.6 ± 0.23 | 2.1 ± 0.2 | 0.001 |
| Resting membrane potential, mV (10) | -50.0 ± 1.2 | -51.0 ± 1. | 0.5411 |
| Cell capacitance, pF (10) | 28.0 ± 1.8 | - | - |
| Threshold for action potential, mV (10) | -32.3 ± 3.7 | -37.6 ± 1.3 | 0.14 |
| Amplitude of action potential, mV (10) | 59.5 ± 6.89 | 61.9 ± 2.9 | 0.746 |
| Cells spontaneously firing action potentials, % (10) | 90 | 100 | - |
| Input resistance, MΩ (10) | 0.4 ± 0.01 | 0.4 ± 0.01 | 0.114 |
| Membrane time constant, ms (10) | 48.57 ± 6.1702 | 41.42 ± 2.7478 | 0.257 |
| AHP time constant, ms | 73 ± 9 | 60 ± 6 | 0.0185 |

Table 7

Summary of basic membrane characteristics of noradrenergic LC neurons that responded with a decrease in their spontaneous firing rate following the application of the V1a antagonist ((d(CH2)51, Tyr(Me)2, Arg8)-Vasopressin, 30 nM. Data are presented as the mean ± SEM.

| Parameter<br>(N) = number of cells | Baseline | V1a antagonist | *P* (paired Student's *t* test) |
|---|---|---|---|
| Spontaneous firing rate, Hz (6) | 2.0 ± 0.3 | 1.4 ± 0.2 | 0.017 |
| Resting membrane potential, mV (6) | -49.7 ± 0.7 | -48.0 ± 1.1 | 0.0143 |
| Cell capacitance, pF (6) | 27.0 ± 3.9 | - | - |
| Threshold for action potential, mV (6) | -36.2 ± 1.0 | -35.8 ± 1.1 | 0.0251 |
| Amplitude of action potential, mV (6) | 65.5 ± 2.0 | 58.3 ± 2.7 | 0.28 |
| Cells spontaneously firing action potentials, % (6) | 100 | 100 | - |
| Input resistance, MΩ (6) | 0.4 ± 0.02 | 0.4 ± 0.0 | 0.16 |
| Membrane Time constant, ms (6) | 39.2 ± 3.0 | 44.7 ± 5.6 | 0.2425 |
| AHP time constant, ms | 61 ± 4 | 69 ± 10 | 0.5117 |

## References


Cuello, A.C., Milstein, C., Couture, R., Wright, B., Priestley, J.V., and Jarvis, J. (1984). Characterization and immunocytochemical application of monoclonal antibodies against enkephalins. *J Histochem Cytochem* 32, 947-957.

Gunn, B.G., Cunningham, L., Cooper, M.A., Corteen, N.L., Seifi, M., Swinny, J.D., Lambert, J.J., and Belelli, D. (2013). Dysfunctional astrocytic and synaptic regulation of hypothalamic glutamatergic transmission in a mouse model of early-life adversity: relevance to neurosteroids and programming of the stress response. *J Neurosci* 33, 19534-19554.

Kerman, I.A., Akil, H., and Watson, S.J. (2006). Rostral elements of sympatho-motor circuitry: a virally mediated transsynaptic tracing study. *J Neurosci* 26, 3423-3433.

Miyazaki, T., Fukaya, M., Shimizu, H., and Watanabe, M. (2003). Subtype switching of vesicular glutamate transporters at parallel fibre-Purkinje cell synapses in developing mouse cerebellum. *Eur J Neurosci* 17, 2563-2572.

Pfeiffer, F., Simler, R., Grenningloh, G., and Betz, H. (1984). Monoclonal antibodies and peptide mapping reveal structural similarities between the subunits of the glycine receptor of rat spinal cord. *Proc Natl Acad Sci U S A* 81, 7224-7227.

Sato, K., Numata, T., Saito, T., Ueta, Y., and Okada, Y. (2011). V(2) receptor-mediated autocrine role of somatodendritic release of AVP in rat vasopressin neurons under hypo-osmotic conditions. *Sci Signal* 4, ra5.

Villanueva, C., Jacquier, S., and De Roux, N. (2012). DLK1 is a somato-dendritic protein expressed in hypothalamic arginine-vasopressin and oxytocin neurons. *PLoS One* 7, e36134.
